# Potential adverse events associated with sphingosine-1-phosphate (S1P) receptor modulators in patients with multiple sclerosis: an analysis of the FDA adverse event reporting system (FAERS) database

**DOI:** 10.3389/fphar.2024.1376494

**Published:** 2024-05-23

**Authors:** Xiping Yang, Yan Yan, Suyao Liu, Zhiqing Wang, Xia Feng

**Affiliations:** Department of Pharmacy, Affiliated Nanjing Brain Hospital, Nanjing Medical University, Nanjing, China

**Keywords:** S1PR modulators, multiple sclerosis, FAERS, adverse event, important medical event, drug safety

## Abstract

**Objective:**

Sphingosine-1-phosphate receptor (S1PR) modulators have recently attracted increasing attention for the treatment of multiple sclerosis (MS). Despite their preference in the clinic, multiple adverse events (AEs) continue to be reported every year. This study aimed to investigate the potential AEs as well as related important medical events (IMEs) signal associated with S1PR modulators, including fingolimod, siponimod and ozanimod in a real-world study using the FDA Adverse Event Reporting System (FAERS) database.

**Methods:**

All data were collected from the FAERS database, spanning from the fourth quarter of 2010(2010Q4) to the second quarter of 2023 (2023Q2). Potential AE and IME signals of S1PR modulators were identified based on a disproportionality analysis using the reporting odds ratio (ROR), proportional reporting ratio (PRR), and the bayesian confidence propagation neural network of information components (IC).

**Results:**

Overall, 276,436 reports of fingolimod, 20,972 reports of siponimod and 10,742 reports of ozanimod were analyzed from the FAERS database. Among reports, females were more prone to develop AEs (73.71% for females vs. 23.21% for males), and more than 50% of patients suffered from AEs were between 18 and 64 years. Subsequently, we investigated the top 20 AEs associated with the signal strength of S1PR modulators at the preferred term (PT) level, and identified 31 (8 vs. 11 vs. 12, respectively) unlabeled risk signals such as thrombosis, uterine disorder and reproductive system and breast disorders. Furthermore, we discovered that the S1PR modulator reported variations in the possible IMEs, and that the IMEs associated with ocular events were reported frequently. It’s interesting to note that infection and malignancy are prominent signals with both fingolimod and siponimod in the top 20 PTs related to mortality reports.

**Conclusion:**

The present investigation highlights the possible safety risks associated with S1PR modulators. The majority of AEs are generally consistent with previous studies and are mentioned in the prescribing instructions, however, several unexpected AE signals have also been observed. Ozanimod showed the lowest signal intensity and a better safety profile than the other S1PR modulators. Due to the short marketing time of drugs and the limitations of spontaneous reporting database, further research is required to identify potential AEs related to S1PR modulators.

## Introduction

Sphingosine-1-phosphate (S1P) is bioactive, soluble lysophospholipid signaling molecule that plays a critical role in multiple physiological and pathophysiological process. S1P acts as a ligand for a family of specific high-affinity G protein-coupled lipid cell surface receptors (S1PR), which is relevant for the regulation of the immune, cardiovascular system and central nervous system (CNS) (Baldin and Lugaresi, 2020; McGinley and Cohen, 2021; Bravo, Cedeño, Casadevall & Ramió-Torrentà, 2022). S1PR modulators are immunomodulatory drugs that target S1P generation, transport, and degradation may represent novel approaches for the treatment of immune-mediated disease (Kunkel, Maceyka, Milstien & Spiegel, 2013). Currently, there are four effective S1PR modulators (fingolimod, siponimod, ozanimod, and ponesimod) with regulatory approval to treat multiple sclerosis (MS). Fingolimod was the first S1PR modulator to gain regulatory approval for the treatment of relapsing-remitting MS (RRMS), which has broad receptor affinity for S1PR1, 3, 4, and 5. Increasing evidence suggests that the use of fingolimod in MS may increase the risk of cardiovascular events such as first-degree AV block and sinus bradycardia (Gold et al., 2014; Ziemssen et al., 2019). The cardiac events usually transient and self-limiting in clinical trials, however, a case of sudden unexpected death was reported in the 5^th^ month of fingolimod treatment in a 48-year-old patient; autopsy results suggested ventricular arrhythmia as the leading cause of death (Lindsey, Haden-Pinneri, Memon & Buja, 2012).

Although research has shown that the toxicity of the new generation of S1PR modulators has mitigated compared to fingolimod owing to their high receptor affinity, there has been an upward trend in the reports of adverse events (AEs) with the increased use of S1PR modulators recent years (Comi et al., 2019; Kappos et al., 2021). Case of skin cancer (squamous cell carcinoma, melanoma, and basal cell carcinoma) have been linked to fingolimod and ozanimod. Other concerns related to S1PR modulators such as macular oedema, leukopenia (Mochizuki, 2009; Camm, Hla, Bakshi & Brinkmann, 2014; Cantalupo et al., 2017), and progressive multifocal leukoencephalopathy occurs (Berger et al., 2018) are also reported. A potential occurrence of serious adverse events could change how the medications are used in clinical practice and modify each drug’s monitoring plan. However, it is unclear whether S1PR modulators may cause other rare or serious AEs. Therefore, a thorough and in-depth description of important AEs caused by S1PR modulators is essential.

Recently, pharmacovigilance databases have been successfully exploited for early detection of rare AEs and continuous monitoring of marketed medications. The Food and Drug Administration Adverse Event Reporting System (FAERS) is a freely accessible public database housing millions of submitted adverse event reports from healthcare professionals, consumers, manufactures, and other stakeholders spontaneously. Its primary purpose is to facilitate the FDA’s post-marketing safety surveillance on pharmaceutical and biological products. The present study aimed to investigate the potential AEs and important medical events (IMEs) signals associated with S1PR modulators using the FAERS database, and to determine whether the use of different S1PR modulators for patients with MS increased the risk of reporting adverse reactions.

## Materials and methods

### Data source and collection

**FIGURE 1:**
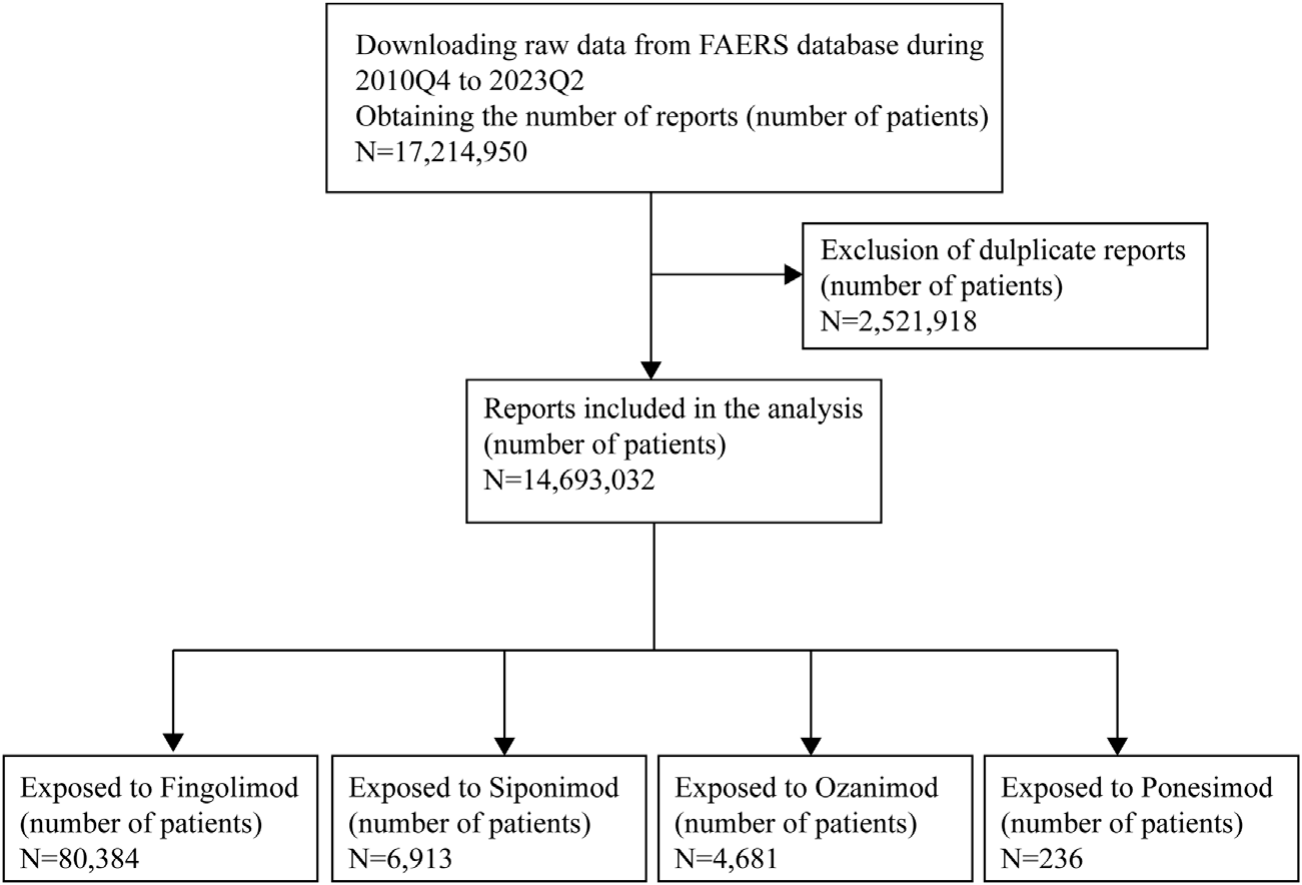
Flowchart of data collection process.

In this study, we conducted a retrospective pharmacovigilance analysis by querying data from the FAERS database covering the period from the fourth quarter of 2010 (2010Q4) to the second quarter of 2023 (2023Q2). The database included seven data files, namely, patient demographic information (DEMO), drug/biologic information (DRUG), adverse events (REAC), patient outcomes (OUTC), report sources (RPSR), start/end dates of drug therapy (THER), and indications for drug (INDI) (Shu, Ding, Liu & Zhang, 2023). In the FAERS database design, a relation was created to connect each data file using some special identification numbers (such as CASEID, PRIMARYID). To ensure report uniqueness, deduplication process should be performed prior to statistical analysis because multiple versions of a report would be reported. The CASEID and the PRIMARYID served as the key filters in our study to remove duplicate records according to the following principles: selecting the latest FDA_DT when the CASEID were the same and selecting the higher PRIMARYID when the CASEID and FDA_DT were the same. Generic names and brand names were applied to identify S1PR modulators-related reports due to two variables, PROD_AI and DRUGNAME (Table 1). The data of ponesimod was available in Supplementary Material. To improve the reported association between drug and AEs, only reports that identified the drugs as primary suspect were selected. All AEs were coded using the preferred terms (PTs) from the Medical Dictionary for Regulatory Activities (MedDRA)n (English version 26.0), and these PTs were categorized into their primary system organ classes (SOCs) in the MedDRA. Two or more PTs reported in one report were considered as different AEs. Serious AEs (SAEs) were classified as death, life-threatening, hospitalization, congenital anomaly, or required intervention to prevent. Figure 1 presents the main collection process.

**TABLE 1:** Summary of FDA-approved S1PR modulators.

Drug name	Brand name	Multiple sclerosis population	Approval year
Fingolimod	Gilenya	RRMS/PPMS	2010
Siponimod	Mayzent	SPMS	2019
Ozanimod	Zeposia	RRMS	2020
Ponesimod	Ponvory	RRMS	2021

Abbreviations: FDA, US, food and drug administration; S1PR, sphingosine-1-phosphat receptor.

### Data analysis

Descriptive analysis was carried out to summarize the clinical characteristic profiles of AE reports associated with S1PR modulators (i.e., gender, age, reporting country, reporting year, and type of reporter). Based on the disproportionality analysis using a 2 × 2 contingency table, the reporting odds ratio (ROR), the proportional reporting ratio (PRR) and Bayesian confidence propagation neural network of information components (IC) were employed to detect signal strength of reports of S1PR modulators at both PT and SOC levels in FAERS database. A potential AE of clinical relevance related to the use of each S1PR modulator was defined when at least one of the three indices met the criteria described as shown in Supplementary Table S1. The proportion of the SOC was calculated as the number of events at the SOC level divided by the total number of events associated with each S1PR modulators. Furthermore, the important medical event terms (IMEs) were listed to identify the potentially important medical AEs based on their seriousness and clinical importance. To further assess the safety of subgroups, we grouped subjects according to demographic characteristics such as age and racial/ethnic analyzed the data separately. SAS version 9.4 (SAS Institute, Cary, NC, United States), Microsoft EXCEL 2019, and the GraphPad Prism 8 (GraphPad Software, CA, USA) were used to compile and process the data.

## Results

### Descriptive analysis

As shown in Figure 1, during the study period, a total of 17,214,950 AE reports were recorded in the FAERS database. Following exclusion of cases without reported primary suspected drug and deduplication process, 80,384 patients of fingolimod, 6,913 patients of siponimod, 4,681 patients of ozanimod and 236 patients of ponesimod were finally retained, respectively. For ponesimod, owing to its short marketing time, there were very few reports suspected to be related to this drug. Thus, we conducted a preliminary study of ponesimod and analyzed the data separately, related information is available in the Supplementary Material. Table 2 depicts the clinical characteristics of patients in AEs. According to the results, in the reports with known indication information, approximately 100% indications of fingolimod and siponimod analyzed in this study were multiple sclerosis, while the proportion of ozanimod was around 75%, which was also in accordance with the clinical practice. Among all reports, females accounted for a larger proportion than males (74.14% vs. 20.87%, 71.47% vs. 22.20%, and 69.58% vs. 26.06%, respectively). Patients were mainly aged 18–64 years (51.70%), with a median age of 44.00 (IQR 35-53), 55.00 (48-62), and 49.00 (38-58) years, respectively. Additionally, most reports came from the United States (63.78%, 78.66%, and 92.31%, respectively). The number of cases peaked in 2019 (fingolimod: 10,395, 12.93%), and 2021 (siponimod: 1848, 26.73%; ozanimod: 1952, 41.70%). Reports from healthcare professionals made up 37.18% of reports. For fingolimod, a total of 45,983 serious outcomes (57.20%) were reported with 1,053 deaths. Siponimod and ozanimod had a total of 3,390 and 1,720 SAE reports, respectively, with majority of them being hospitalizations or others.

**TABLE 2:** Clinical characteristics of patients with AEs associated with S1PR modulators in FAERS database (2010Q4 to 2023Q2).

	Fingolimod	Siponimod	Ozanimod	Total
Number of reports	80,384	6,913	4,681	91,978
Gender, n (%)				
Male	16,776 (20.87)	1,535 (22.20)	1,220 (26.06)	19,531 (21.23)
Female	59,600 (74.14)	4,941(71.47)	3,257 (69.58)	67,798 (73.71)
Unknown	4,008 (4.99)	437 (6.32)	204 (4.36)	4,649 (5.05)
Age, years				
Median (interquartile)	44.00 (35-53)	55.00 (48-62)	49.00 (38-58)	
<18, n (%)	636 (0.79)	6 (0.09)	4 (0.09)	646 (0.70)
18-44, n (%)	22,086 (27.48)	532 (7.70)	1,420 (30.34)	24,038 (26.13)
45-64, n (%)	19,943 (24.81)	1918 (27.74)	1,660 (35.46)	23,521 (25.57)
65-74, n (%)	1716 (2.13)	392 (5.67)	366 (7.82)	2,474 (2.69)
≥75, n (%)	120 (0.15)	59 (0.85)	71 (1.52)	250 (0.27)
Unknown, n (%)	35,883 (44.64)	4,006 (57.95)	1,160 (24.78)	41,049 (44.63)
Reporting country, n (%)				
United States	51,270 (63.78)	5,438 (78.66)	4,321 (92.31)	61,029 (66.35)
Other/Unknown	29,114 (36.22)	1,475 (21.34)	360 (7.69)	30,949 (33.65)
Reporting year, n (%)				
2010 (October-December)	21 (0.03)	-	-	21 (0.02)
2011	2,174 (2.70)	-	-	2,174 (2.36)
2012	3,229 (4.02)	-	-	3,229 (3.51)
2013	7,818 (9.73)	-	-	7,818 (8.50)
2014	2,970 (3.69)	-	-	2,970 (3.23)
2015	6,510 (8.10)	-	-	6,510 (7.08)
2016	8,981 (11.17)	-	-	8,981 (9.76)
2017	9,112 (11.34)	-	-	9,112 (9.91)
2018	10,338 (12.86)	-	-	10,338 (11.24)
2019	10,395 (12.93)	683 (9.88)	-	11,078 (12.04)
2020	8,588 (10.68)	1805 (26.11)	214 (4.57)	10,607 (11.53)
2021	4,504 (5.60)	1848 (26.73)	1952 (41.70)	8,304 (9.03)
2022	4,061 (5.05)	1,658 (23.98)	1752 (37.43)	7,471 (8.12)
2023 (January-June)	1,683 (2.09)	919 (13.29)	763 (16.30)	3,365 (3.66)
Type of reporter, n (%)				
Consumer	50,611 (62.96)	4,367 (63.17)	1,604 (34.27)	56,582 (61.52)
Healthcare professional	28,781 (35.80)	2,351 (34.01)	3,061 (65.39)	34,193 (37.18)
Other/unknown	992 (1.23)	195 (2.82)	16 (0.34)	1,203 (1.31)
Outcome, n (%)				
Death	1,053 (1.31)	151 (2.18)	52 (1.11)	1,256 (1.37)
Life-threatening	1,121 (1.39)	69 (1.00)	48 (1.03)	1,238 (1.35)
Hospitalization	10,146 (12.62)	980 (14.18)	450 (9.61)	11,576 (12.59)
Disability	1,140 (1.42)	63 (0.91)	68 (1.45)	1,271 (1.38)
Congenial anomaly	138 (0.17)	0 (0.00)	1 (0.02)	139 (0.15)
Required intervention	23 (0.03)	4 (0.06)	0 (0.00)	27 (0.03)
Other serious	32,362 (40.26)	2,123 (30.71)	1,101(23.52)	34,485 (37.49)
Indication, n (%)				
Multiple sclerosis	64,525 (80.27%)	4,668 (67.52%)	2,978 (63.62)	72,172 (78.47%)
Ulcerative colitis	0	0	924 (19.74)	924 (1.00%)
Others	547 (0.68%)	56 (0.82%)	120 (2.56%)	722 (0.78%)
Unknown	15,312 (19.05)	2,189 (31.66)	659 (14.08)	18,160 (19.74)

### Comprehensive AE signal analysis overall and by subgroups

**FIGURE 2:**
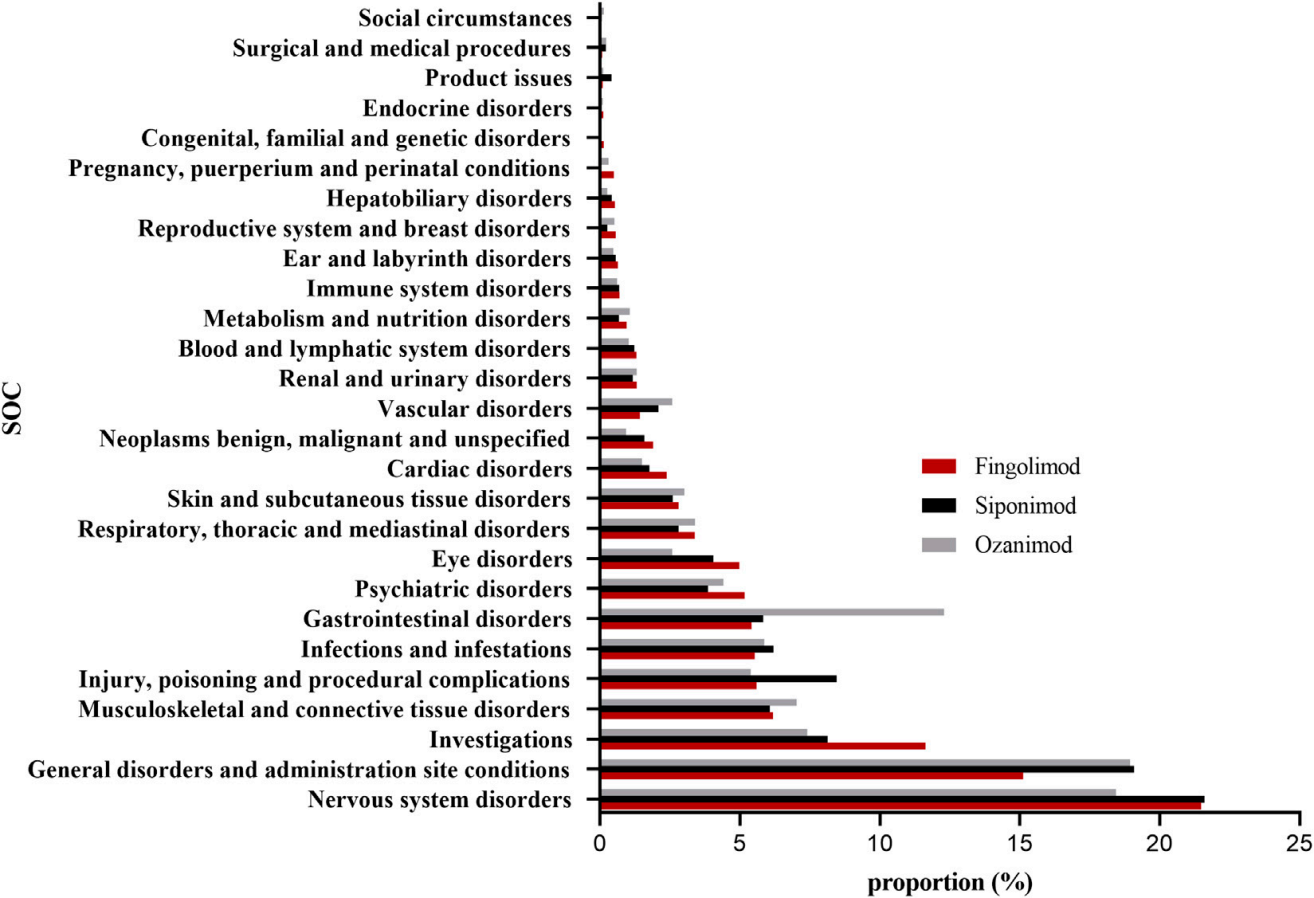
Proportion of S1PR modulators-related AEs at the System Organ Class (SOC) level. AEs, adverse events; S1PR, Sphingosine-1-phosphate receptor.

Figure 2; Supplementary Table S2 presented the proportions of SOCs for a total of 276,436, 20,972, and 10,742 potential AEs of fingolimod, siponimod and ozanimod, respectively. As SOCs terms, general disorders and administration site were highest both in fingolimod and siponimod (21.48% and 21.60%, respectively), while nervous system disorders exhibiting remarkable association with ozanimod treatment (18.94%). Next, we compared the AE reports for fingolimod, siponimod and ozanimod at the SOC level by subgroup analysis. Table 3 displays the number of AE reports for pediatric MS patients (POMS; <18 years) and late-onset MS patients (LOMS; >50 years) during the study period. Stratified analysis by age demonstrated that both LOMS and POMS patient treatment with fingolimod revealed significant signals for “eye disorders”, “nervous system disorders”, “investigations”, and “musculoskeletal and connective tissue disorders”. In addition, positive signals associated with “ear and labyrinth disorders” and “psychiatric disorders” were identified in LOMS patients, while “pregnancy, puerperium and perinatal conditions” was only found in the POMS group. Siponimod and ozanimod showed similar significant signals for LOMS patients compared to fingolimod. Meanwhile, we also observed strong signal related to vascular disorders in LOMS patients administrated with ozanimod. Understanding ethnic and geographical differences in adverse reactions have attracted increasing scientific interest. Due to the lack of detailed ethnic information, we used the broad geographic categorization of “racial” group in our study, and categorized race and ethnicity as White (European-American), Black (African-American), Asian, and other or unknown race and ethnicity. As demonstrated in Supplementary Table S3, both fingolimod and siponimod considerably increased the reporting probability of “nervous system disorders” among all ethnicities. Similar risk has been observed in White and Black patients taking ozanimod. However, infections were found to be more likely occurred in Asians treatment with ozanimod.

**TABLE 3:** Comparison of the AE reports of S1PR modulators at the system organ class (SOC) level by age group.

SOC	Fingolimod				Siponimod		Ozanimod	
	<18 years		>50 years		>50 years		>50 years	
	N	ROR	N	ROR	N	ROR	N	ROR
Blood and lymphatic system disorders	38	0.63	687	0.56	95	0.57	31	0.29
Cardiac disorders	60	1.27	1,676	0.93	180	0.72	78	0.49
Congenital, familial and genetic disorders	38	0.95	21	0.44	6	0.92	0	—
Ear and labyrinth disorders	11	1.70	389	1.42	41	1.09	33	1.39
Endocrine disorders	1	0.12	81	0.49	6	0.27	6	0.42
Eye disorders	127	3.19	3,140	2.79	364	2.34	154	1.54
Gastrointestinal disorders	156	0.90	3,479	0.60	511	0.65	694	1.52
General disorders and administration site conditions	319	0.98	8,861	0.86	1,419	1.04	1,019	1.22
Hepatobiliary disorders	5	0.18	276	0.47	31	0.39	19	0.38
Immune system disorders	20	0.56	387	0.63	44	0.52	38	0.72
Infections and infestations	129	0.87	3,623	1.01	518	1.06	296	0.95
Injury, poisoning and procedural complications	229	0.56	3,361	0.55	640	0.79	296	0.57
Investigations	241	1.90	7,292	1.98	654	1.24	303	0.89
Metabolism and nutrition disorders	25	0.44	641	0.40	64	0.30	50	0.37
Musculoskeletal and connective tissue disorders	117	2.02	4,050	1.16	550	1.15	387	1.29
Neoplasms benign, malignant and unspecified (incl cysts and polyps)	12	0.59	1,681	0.87	214	0.81	50	0.29
Nervous system disorders	558	3.35	11,588	2.66	1728	2.96	884	2.28
Pregnancy, puerperium and perinatal conditions	64	2.83	—	—	—	—	—	—
Product issues	3	0.05	66	0.09	35	0.36	11	0.18
Psychiatric disorders	81	0.47	2,764	1.16	320	0.98	201	0.97
Renal and urinary disorders	11	0.32	863	0.62	113	0.59	74	0.62
Reproductive system and breast disorders	18	0.73	172	0.78	18	0.60	13	0.68
Respiratory, thoracic and mediastinal disorders	113	0.96	2,232	0.65	248	0.53	206	0.70
Skin and subcutaneous tissue disorders	83	0.34	1777	0.58	205	0.49	163	0.62
Social circumstances	0	—	32	0.15	4	0.14	9	0.48
Surgical and medical procedures	1	0.05	58	0.07	23	0.21	23	0.33
Vascular disorders	23	0.53	1,035	0.72	185	0.95	153	1.25

We further detected the top 20 PTs ordered by signal strength referring to ROR, PRR and BCPNN analysis, and the results are shown in Table 4. Among them, we identified some PTs that were not recorded in the instructions of S1PR modulators as follows: 8 AEs for fingolimod, including hemihyperaesthesia, haemorrhagic adrenal infarction, vibration test abnormal, hoffmann’s sign, lentigo, lhermitte’s sign, tandem gait test abnormal, and romberg test positive; 11 AEs for siponimod lipids, including decreased, bowen’s diseaseurinary, urinary tract inflammation, gait spastic, hypertensive, atrioventricular, uhthoff’s phenomenon, paraparesis, dysmetria, band sensation, and amblyopia; 12 AEs for ozanimod, including uterine disorder, head titubation, penile swelling, auditory disorder, sleep deficit, brain fog, papillary thyroid cancer, peroneal nerve palsy, lymph node pain, periorbital swelling, and bladder disorder.

**TABLE 4:** The signal strength of the top 20 adverse events (AEs) of S1PR modulators.

SOC	PT	N	PRR (χ^2^)	ROR (95% CI)	IC
Fingolimod					
Nervous system disorders	Hemihyperaesthesia	3	154.16 (228.26)	154.17 (31.12-763.85)	6.28
Endocrine disorders	Haemorrhagic adrenal infarction	4	102.78 (241.89)	102.78 (29.00-364.22)	5.96
Infections and infestations	Osseous cryptococcosis^a^	3	66.07 (134.58)	66.07 (17.08-255.51)	5.54
Investigations	Vibration test abnormal	8	61.67 (341.04)	61.67 (27.16-140.01)	5.47
Investigations	JC polyomavirus test positive^a^	449	58.41 (18,375.62)	58.51 (52.48-65.22)	5.41
Investigations	CD30 expression^a^	10	53.16 (380.56)	53.16 (25.91-109.08)	5.31
Investigations	Lymphocyte count decreased^a^	3,411	53.03 (129,602.68)	53.68 (51.62-55.82)	5.31
Nervous system disorders	Hoffmann’s sign	11	49.88 (398.08)	49.88 (25.27-98.44)	5.25
Nervous system disorders	Gait spastic	64	48.60 (2,268.70)	48.61 (36.71-64.39)	5.22
Investigations	ECG signs of myocardial infarction^a^	4	47.44 (139.04)	47.44 (15.47-145.48)	5.19
Eye disorders	Macular oedema^a^	865	47.27 (29,986.50)	47.42 (43.93-51.17)	5.19
Nervous system disorders	Central nervous system lesion^a^	2,739	46.86 (94,300.08)	47.32 (45.33-49.39)	5.18
Investigations	Dermatologic examination abnormal^a^	3	46.25 (102.16)	46.25 (12.73-168.05)	5.16
Skin and subcutaneous tissue disorders	Lentigo	69	45.27 (2,308.91)	45.28 (34.62-59.22)	5.14
Infections and infestations	Cryptococcal cutaneous infection^a^	22	45.22 (735.59)	45.22 (28.12-72.74)	5.14
Neoplasms benign, malignant and unspecified	Haemangioma of skin^a^	65	43.95 (2,123.02)	43.96 (33.37-57.91)	5.11
Nervous system disorders	Lhermitte’s sign	50	43.80 (1,628.38)	43.80 (32.00-59.97)	5.10
Investigations	Tandem gait test abnormal	12	40.22 (363.95)	40.22 (21.31-75.92)	5.00
Neoplasms benign, malignant and unspecified	Fibrous histiocytoma^a^	50	38.73 (1,468.95)	38.74 (28.41-52.83)	4.96
Investigations	Romberg test positive	25	38.54 (731.34)	38.54 (24.87-59.75)	4.96
Siponimod					
Investigations	Lipids decreased	3	125.16 (348.18)	125.18 (39.01-401.63)	6.88
Investigations	Lymphocyte count^a^ decreased	307	48.20 (13,867.67)	48.90 (43.63-54.81)	5.56
Investigations	Lymphocyte count abnormal^a^	16	45.24 (677.21)	45.27 (27.58-74.31)	5.47
Blood and lymphatic system disorders	Lymphopenia^a^	142	30.70 (4,021.22)	30.91 (26.17-36.50)	4.92
Neoplasms benign, malignant and unspecified (incl cysts and polyps)	Bowen’s disease^a^	13	29.79 (356.55)	29.81 (17.24-51.55)	4.88
Eye disorders	Macular oedema^a^	51	28.68 (1,343.79)	28.75 (21.80-37.91)	4.82
Renal and urinary disorders	Urinary tract inflammation	3	24.63 (67.20)	24.63 (7.89-76.90)	4.61
Nervous system disorders	Gait spastic	3	23.23 (63.10)	23.23 (7.44-72.51)	4.52
Vascular disorders	Hypertensive urgency^a^	3	22.55 (61.10)	22.55 (7.23-70.36)	4.48
Nervous system disorders	Paresis	16	22.25 (321.24)	22.27 (13.60-36.45)	4.46
Cardiac disorders	Atrioventricular block first degree^a^	29	20.46 (531.58)	20.49 (14.21-29.55)	4.34
Nervous system disorders	Uhthoff’s phenomenon	6	20.01 (107.30)	20.01 (8.96-44.73)	4.31
Nervous system disorders	Paraparesis	11	19.20 (188.04)	19.21 (10.61-34.79)	4.25
Nervous system disorders	Dysmetria	4	18.17 (64.33)	18.17 (6.79-48.64)	4.17
Nervous system disorders	Band sensation	9	17.21 (136.28)	17.22 (8.93-33.19)	4.09
Nervous system disorders	Electric shock sensation	10	17.21 (151.39)	17.22 (9.24-32.08)	4.09
Nervous system disorders	Trigeminal neuralgia^a^	26	17.20 (393.31)	17.22 (11.70-25.33)	4.09
Eye disorders	Amblyopia	3	16.85 (44.36)	16.85 (5.41-52.50)	4.06
Investigations	Heart rate decreased^a^	179	14.62 (2,256.09)	14.74 (12.71-17.08)	3.86
Investigations	JC polyomavirus test positive^a^	11	13.86 (130.32)	13.86 (7.66-25.08)	3.78
Ozanimod					
Investigations	Lymphocyte count abnormal^a^	7	38.17 (251.01)	38.20 (18.14-80.43)	5.24
Blood and lymphatic system disorders	Lymphopenia^a^	48	20.07 (865.54)	20.15 (15.17-26.78)	4.32
Investigations	Lymphocyte count decreased^a^	66	19.87 (1,177.14)	19.98 (15.68-25.47)	4.31
Reproductive system and breast disorders	Uterine disorder	6	14.26 (73.70)	14.26 (6.40-31.80)	3.83
Nervous system disorders	Head titubation	3	12.53 (31.72)	12.53 (4.03-39.93)	3.64
Reproductive system and breast disorders	Penile swelling	3	12.37 (31.26)	12.38 (3.98-38.44)	3.62
Eye disorders	Macular oedema^a^	11	11.95 (110.04)	11.96 (6.62-21.62)	3.57
Ear and labyrinth disorders	Auditory disorder	3	11.92 (29.91)	11.92 (3.84-37.03)	3.57
Cardiac disorders	Cardiac flutter^a^	13	11.70 (126.80)	11.71 (6.79-20.19)	3.54
Nervous system disorders	Optic neuritis	17	10.63 (147.94)	10.65 (6.61-17.14)	3.41
Nervous system disorders	Sleep deficit	3	10.60 (26.01)	10.60 (3.41-32.92)	3.40
Nervous system disorders	Brain fog	3	10.01 (24.28)	10.02 (3.23-31.10)	3.32
Neoplasms benign, malignant and unspecified	Papillary thyroid cancer	3	10.00 (24.23)	10.00 (3.22-31.05)	3.32
Investigations	Heart rate decreased^a^	62	9.84 (491.55)	9.89 (7.70-12.70)	3.30
Investigations	JC polyomavirus test positive^a^	4	9.80 (31.52)	9.80 (3.67-26.15)	3.29
Nervous system disorders	Peroneal nerve palsy	10	9.64 (77.28)	9.65 (5.19-17.95)	3.27
Blood and lymphatic system disorders	Lymph node pain	3	9.17 (21.79)	9.17 (2.95-28.48)	3.19
Immune system disorders	Multiple allergies^a^	12	8.71 (81.77)	8.72 (4.95-15.37)	3.12
Eye disorders	Periorbital swelling	6	8.31 (38.49)	8.31 (3.73-18.52)	3.05
Renal and urinary disorders	Bladder disorder	14	7.84 (83.37)	7.85 (4.64-13.26)	2.97

^a^ Adverse events mentioned in the instructions of fingolimod, siponimod and ozanimod, respectively.

### Analysis of important medical events (IMEs)

**FIGURE 3:**
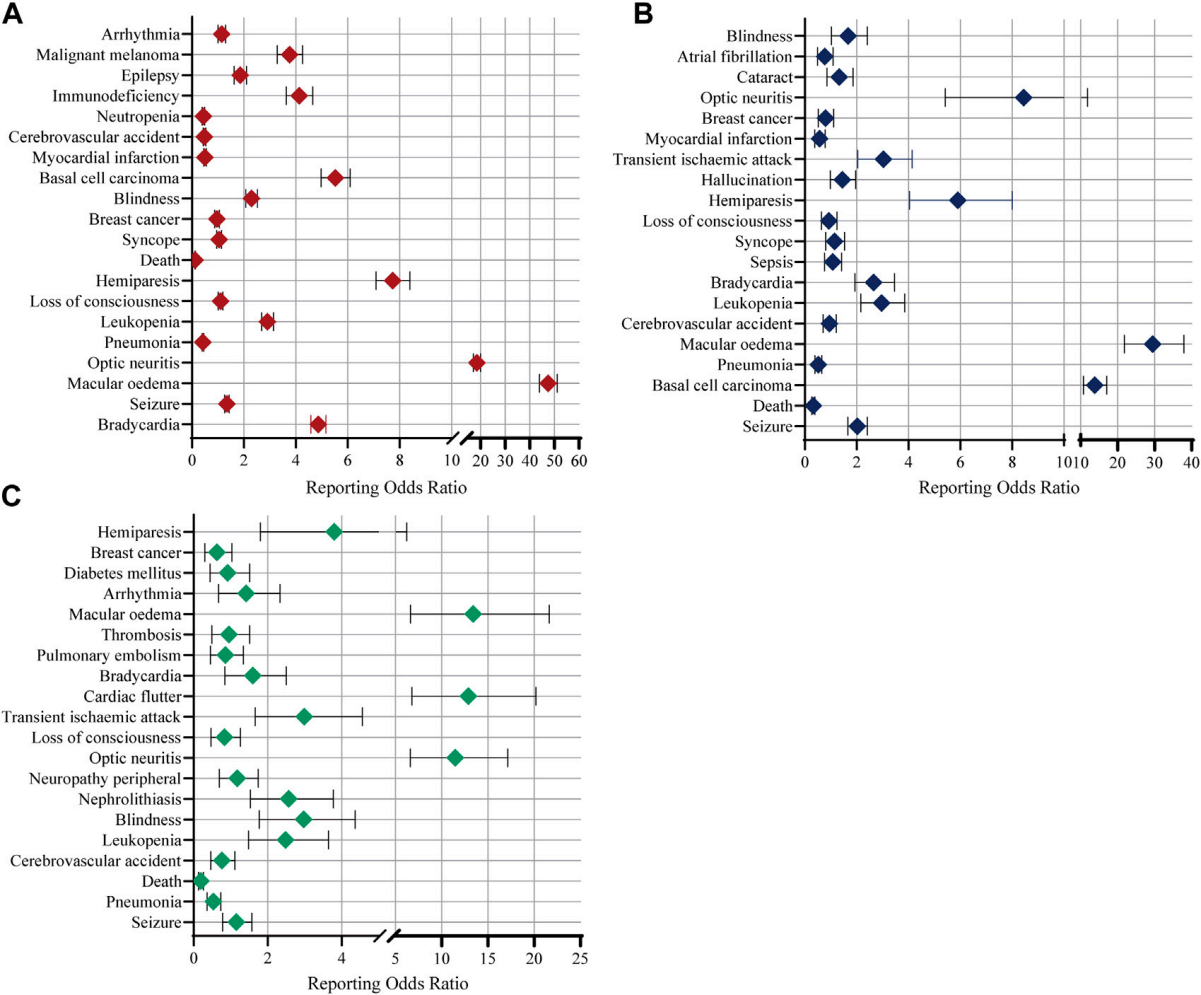
Reporting odds ratios (RORs) of the top 20 IMEs associated with S1PR modulators. Colours represent different drugs: **(A)** Fingolimod; **(B)** Siponimod; **(C)** Ozanimod. Error bars represent the 95% confidence interval (CI). A lower limit of the ROR 95% CI above 1 was considered significant. IMEs, important medical events; S1PR, Sphingosine-1-phosphate receptor.

Data in Supplementary Table S4 displays the top 20 possible important medical events (IMEs) derived from the FAERS database. Analysis indicates that fingolimod, siponimod and ozanimod were associated with cardiac disorders (e.g., bradycardia), nervous system disorders (e.g., seizure, cerebrovascular accident, optic neuritis, loss of consciousness and hemiparesis), infections (e.g., pneumonia), blood and lymphatic system disorders (e.g., leukopenia), eye disorders (e.g., blindness) and breast cancer. Apart from that, syncope, basal cell carcinoma and myocardial infarction was specified for fingolimod and siponimod, while nephrolithiasis and pulmonary embolism was indicated in ozanimod. There have been reports of malignant melanoma and epilepsy with fingolimod, as well as sepsis and atrial fibrillation with siponimod. Additionally, vascular disorders (e.g., thrombosis) as well as metabolism and nutrition disorders (e.g., diabetes mellitus) were also reported. Figure 3 show the signal strength according to ROR. In general, this frequency was much higher for fingolimod than for siponimod and ozanimod, which could be due to the shorter marketing time and improved safety of siponimod and ozanimod.

**FIGURE 4:**
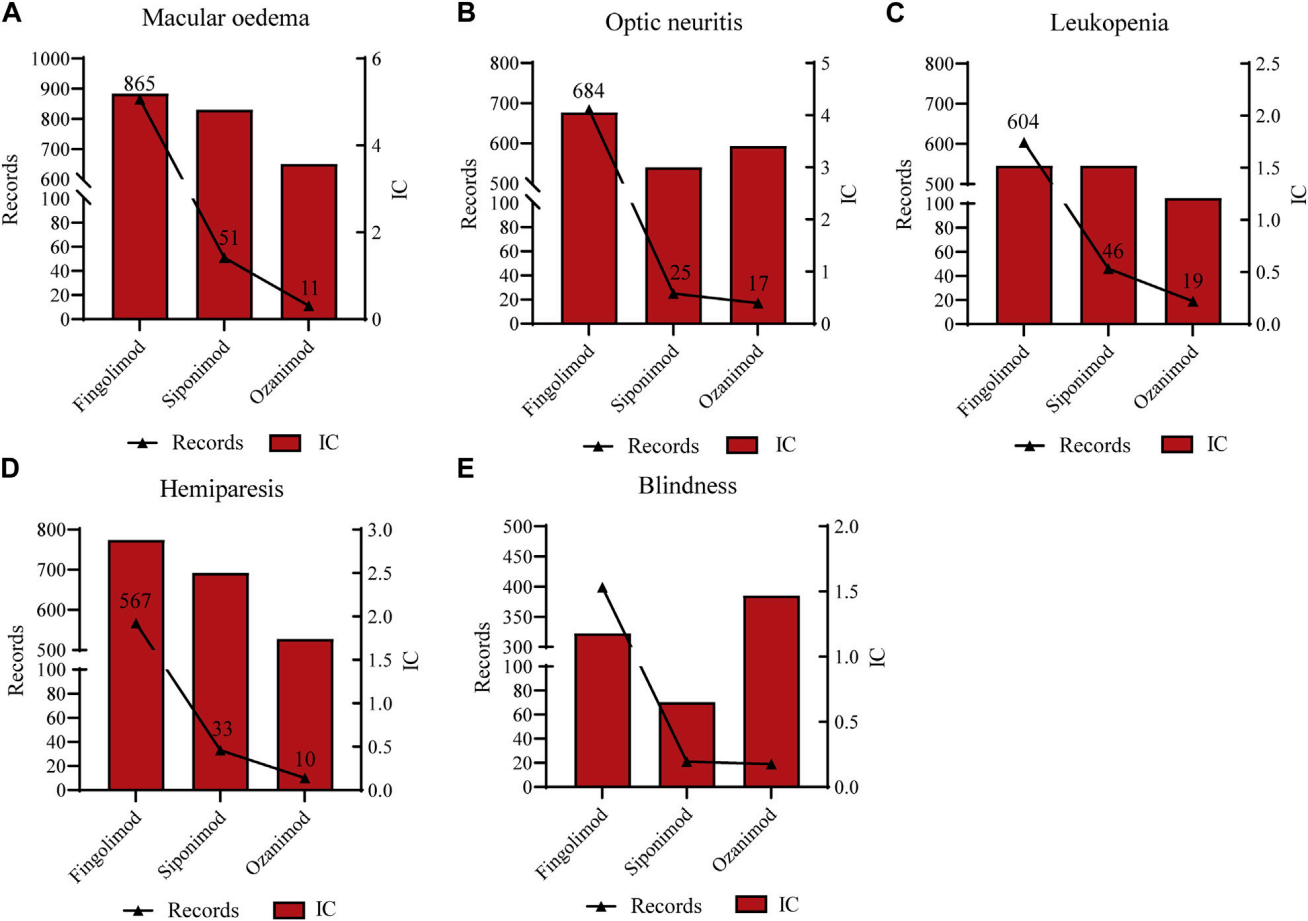
Associations between the five common strong-signal PTs and S1PR modulators quantified by IC values. **(A)** Macular oedema; **(B)** Optic neuritis; **(C)** Leukopenia; **(D)** Hemiparesis; **(E)** Blindness.

As shown in Supplementary Table S4, macular oedema (865, 51, 11, respectively), optic neuritis (684, 25, 17, respectively), leukopenia (604,46,19, respectively), hemiparesis (567,33,10, respectively) and blindness (399, 21, 19, respectively) were the most common significant signals for all the S1PR modulators. Further investigation of the relationships between the five common strong-signal PTs and S1PR modulators quantified by IC values indicated that: macular oedema and optic neuritis were strongly associated with all the S1PR modulators (fingolimod: corresponding IC = 5.19 and 4.05; siponimod: corresponding IC = 4.82 and 3.00; ozanimod: corresponding IC = 3.57 and 3.41, respectively) (Figure 4). The findings mentioned above suggested that fingolimod and siponimod may be responsible for the development of leukopenia and hemiparesis, whereas blindness was strongly associated with ozanimod.

### Fatalities due to S1PR modulators associated adverse events

Due to the low selectivity of fingolimod, serious adverse events have occurred; therefore, its clinical application has been severely restricted. Of note, the majority of adverse events (AEs) caused by ozanimod were mild to moderate in severity and were well-tolerated in patients with multiple sclerosis. Thus, we further analyzed and compared the potential death-related AEs between fingolimod and siponimod. As shown in Table 5, In addition to infections such as progressive multifocal leukoencephalopathy (PML), cryptococcal meningoencephalitis and neuro cryptococcosis, neoplasms benign, malignant and unspecified were an important death cause with fingolimod, whereas cardiac disorders were critical cause of death with siponimod. Besides, both fingolimod and siponimod have been observed to have strong signals in PML.

**TABLE 5:** The signal strength of the top 20 AEs of fingolimod and siponimod according to death reports.

SOC	PT	N	PRR (χ^2^)	ROR (95% CI)	IC
Fingolimod					
Eye disorders	Macular oedema	865	47.27 (29,986.50)	47.42 (43.93-51.17)	5.19
Infections and infestations	Cryptococcal meningoencephalitis	12	32.46 (302.21)	32.46 (17.42-60.49)	4.75
Investigations	CSF protein abnormal	3	22.02 (52.68)	22.02 (6.57-73.84)	4.28
Infections and infestations	Neurocryptococcosis	4	21.26 (67.88)	21.26 (7.48-60.49)	4.23
Investigations	Electrocardiogram PR shortened	15	19.27 (230.97)	19.27 (11.27-32.96)	4.11
Investigations	CSF glucose decreased	6	19.27 (92.39)	19.27 (8.25-45.03)	4.11
Nervous system disorders	Optic neuritis	684	18.44 (10,080.83)	18.49 (17.08-20.02)	4.05
Neoplasms benign, malignant and unspecified	Anaplastic large cell lymphoma T- and null-cell types	13	17.13 (177.70)	17.13 (9.66-30.38)	3.96
Nervous system disorders	Paraparesis	111	15.98 (1,412.17)	15.98 (13.15-19.43)	3.87
Nervous system disorders	Brain stem syndrome	31	12.45 (301.93)	12.45 (8.63-17.95)	3.53
Infections and infestations	JC virus infection	77	12.33 (742.09)	12.33 (9.78-15.55)	3.52
Infections and infestations	Meningitis cryptococcal	73	11.93 (678.79)	11.94 (9.41-15.15)	3.48
Renal and urinary disorders	Neurogenic bladder	97	11.36 (853.86)	11.37 (9.25-13.97)	3.41
Investigations	CSF protein increased	25	10.28 (196.29)	10.28 (6.86-15.41)	3.28
Eye disorders	Ophthalmoplegia	76	9.41 (538.45)	9.41 (7.47-11.87)	3.16
Nervous system disorders	Neuromyelitis optica spectrum disorder	51	9.29 (356.01)	9.30 (7.01-12.33)	3.14
Neoplasms benign, malignant and unspecified	Penile squamous cell carcinoma	3	9.07 (20.34)	9.07 (2.83-29.06)	3.11
Neoplasms benign, malignant and unspecified	Squamous cell carcinoma of the cervix	8	9.00 (53.77)	9.00 (4.41-18.36)	3.10
Nervous system disorders	Hemiparesis	567	7.70 (3,149.19)	7.71 (7.09-8.39)	2.88
Nervous system disorders	Demyelination	118	7.52 (636.09)	7.52 (6.25-9.05)	2.85
Siponimod					
Infections and infestations	JC virus infection	3	5.91 (12.21)	5.91 (1.90-18.37)	2.56
Nervous system disorders	Hemiparesis	33	5.68 (126.79)	5.68 (4.04-8.00)	2.50
Infections and infestations	Urosepsis	12	3.84 (25.22)	3.85 (2.18-6.78)	1.94
Cardiac disorders	Bradycardia	45	2.58 (43.42)	2.58 (1.93-3.46)	1.36
Nervous system disorders	Paralysis	12	2.54 (11.15)	2.54 (1.44-4.47)	1.34
Infections and infestations	Pyelonephritis	7	2.45 (5.98)	2.45 (1.17-5.13)	1.29
Nervous system disorders	Seizure	109	1.99 (54.01)	2.00 (1.66-2.41)	0.99
Nervous system disorders	Status epilepticus	7	1.92 (3.08)	1.92 (0.91-4.03)	0.94
Infections and infestations	COVID-19 pneumonia	8	1.85 (3.14)	1.85 (0.93-3.71)	0.89
Vascular disorders	Hypertensive crisis	7	1.79 (2.45)	1.79 (0.85-3.76)	0.84
Psychiatric disorders	Hallucination	32	1.39 (3.44)	1.39 (0.98-1.96)	0.47
Renal and urinary disorders	Nephrolithiasis	17	1.10 (0.15)	1.10 (0.68-1.77)	0.14
Infections and infestations	Sepsis	39	1.04 (0.06)	1.04 (0.76-1.42)	0.05
Cardiac disorders	Acute myocardial infarction	10	1.03 (0.01)	1.03 (0.55-1.91)	0.04
Nervous system disorders	Unresponsive to stimuli	8	0.94 (0.03)	0.94 (0.47-1.88)	−0.09
Nervous system disorders	Cerebrovascular accident	50	0.92 (0.36)	0.92 (0.70-1.21)	−0.12
Nervous system disorders	Loss of consciousness	36	0.89 (0.48)	0.89 (0.64-1.24)	−0.17
Nervous system disorders	Cerebral infarction	7	0.86 (0.17)	0.86 (0.41-1.80)	−0.22
Neoplasms benign, malignant and unspecified	Breast cancer	27	0.76 (1.98)	0.76 (0.52-1.11)	−0.39
Respiratory, thoracic and mediastinal disorders	Acute respiratory distress syndrome	4	0.74 (0.36)	0.74 (0.28-1.97)	−0.43

## Discussion

As a novel targeted therapy, S1PR modulators have changed the landscape of oral disease modifying therapy (DMT) of multiple sclerosis (MS) over the past several years. Real-word experience with second-generation of S1PR modulators is still in its infancy, thus, it is necessary to generate a comprehensive analysis of potential AEs using real-word database. To the best of our knowledge, this is the first study that comprehensively compared the clinically relevant potential AEs for each S1PR modulators using the FAERS database. Our study has provided supportive disproportionality analysis and data on the clinical characteristics of these adverse events.

Relapsing-remitting multiple sclerosis (RRMS) is the most common MS phenotype, affecting nearly 80% of the patients, in which a clinical attack heralds the onset of the disease. Approximately 60%–70% of those with RRMS may conversion to secondary progressive multiple sclerosis (SPMS) in 1–3 decades after disease onset. Primary progressive multiple sclerosis (PPMS) affects 15%–20% of the patients and defined as gradually progressive and unremitting loss of neurological function for more than 1 year (Cree et al., 2021; Dimitriou, Meuth, Martinez-Lapiscina, Albrecht & Menge, 2023). Fingolimod was the first oral S1PR modulator approved for MS, which has been evaluated in patients with PPMS and RRMS. Siponimod is currently the only approved therapy for SPMS. Ozanimod was approved for RRMS in March 2020. Safety data from short-term studies of siponimod, ozanimod and ponesimod are broadly similar to those fingolimod studies (Olsson et al., 2014; Cohen et al., 2016; Kappos et al., 2016). However, cardiac events were reported as reasons for discontinuation of patients receiving siponimod and ponesimod, and second-degree AV block was the only serious AE seen with siponimod (Olsson et al., 2014). With ozanimod, there were no discontinuation owing to an AE (Cohen et al., 2016). Medications that are highly effective in RRMS have failed in the treatment of progressive MS. Meanwhile, there are potential risks associated with progression, including obesity, hypertension and vascular disorders (Moss et al., 2017; Faissner, Plemel, Gold & Yong, 2019).

Multiple sclerosis commonly affects young females at the ages 20–40 years old. Evidence indicates that the median clinical onset of MS is approximately 29 years old, and the ratio of female to male is approaches 3:1 and may be increasing (Wingerchuk and Carter, 2014; Walton et al., 2020). In our study, reports from female patients accounted for 73.7%, and approximately 50% of the patients were at 18–45 years old in the known age information reports, consistent with previous research. There is rising incidence and prevalence of MS in the demographic extremes, such as pediatric-onset MS (POMS; occurring before 18 years of age) and late-onset MS (corresponding to an onset above 50 years) (Capasso et al., 2023). However, the safety and efficiency data of DMTs including several S1PR modulators in older people and children are still lacking (Vaughn et al., 2019; Krysko et al., 2020). Fingolimod has been associated with cases of progressive multifocal leukoencephalopathy (PML), and these seem to have an age-dependent trend (Grebenciucova and Berger, 2017). Additionally, previous study suggested that higher age is a risk factor for other infections: for example, a higher risk of cryptococcal meningitis was observed in older patients receiving fingolimod (Weideman et al., 2017). Consistent with the research, we found strong signals associated with John Cunningham virus (JCV) index in LOMS patient treatment with fingolimod. In addition, vascular disorders in LOMS patients administrated with ozanimod required more attention.

The potential AE signals and types vary for each S1PR modulators owing to their structural differences. Fingolimod is the universal S1PR modulators due to its wide spectrum effect on S1PR1, S1PR3, S1PR4 and S1PR5. This fact also implies that fingolimod has more side effect than other S1PR modulators (Pournajaf, Dargahi, Javan & Pourgholami, 2022). Studies have found that fingolimod may cause undesirable effects because of its interactions with other S1PR subtypes. Based on the natural distribution pattern of S1P-receptor subtypes, the risk of adverse events has notably increased (Mandal, Gupta, Fusi-Rubiano, Keane & Yang, 2017). Siponimod and ozanimod selectively bind to the S1PR1 and S1PR5 subtypes, and most of their effects derive from this linkage. Unlike fingolimod, Ozanimod does not require phosphorylation for activation and it has a 27-fold selectivity for S1PR1 over S1PR5 (Scott et al., 2016; Chun et al., 2021). The lack of interaction with S1PR3 minimizes potential safety concerns. According to a result of network meta-analysis (NMA), Ozanimod possesses an excellent advantage in terms of reducing the ARR and a low adverse reaction rate. Comparably, fingolimod possesses satisfactory therapeutic effects, while it has a higher adverse reaction rate (Tong et al., 2021).

According to our analysis, the most common serious adverse events (SAEs) caused by all the S1PR modulators were associated with ocular events, which was consistent with previous safety data (Khan et al., 2023; Li et al., 2023). Among the related IMEs, macular oedema was reported frequently. S1PR modulators have been observed to have numerous effects on the functionality of the eye. One observational study reported the reduction of neuronal degeneration linked to the consumption of fingolimod in RRMS patients (El et al., 2021). An increase in macular volume was observed in MS patients treated with fingolimod using spectral-domain optical coherence tomography (OCT). A combined group of 30 patients consuming fingolimod for 5 months had a macular volume increase of 0.025 mm^3^, and in total, 74% of the patients who ingested fingolimod exhibited an increased in macular volume vs. 37% of eyes in the comparison group (Nolan et al., 2013). Nearly all the approved MS disease-modifying therapies have demonstrated a treatment effect on brain atrophy, which could result in an increase in retinal volume. Additionally, macular edema is also associated with the fingolimod modulation of S1PRs on the action of tight junctions of the blood-brain barrier and neurons (Khan et al., 2023). Coppes et al. reported a patient clinically presented with bilateral blurred central vision, painless, and no eye pain with eye movement after 10 days of fingolimod treatment (Coppes, Gutierrez, Reder, Ksiazek & Bernard, 2013). The OCT revealed bilateral macular edema and an accumulation of fluid between the inner nuclear layer and outer plexiform layer. Interestingly, later OCT exams showed a progressive decrease in central foveal thickness and macular volume, without specific treatment other than discontinuation of fingolimod. The underlying mechanism is unknown, but is hypothesized to be increased vascular permeability mediated by S1PR1 and S1PR3 on endothelial cells, which alters barrier function (Zhang et al., 2022). Macular edema cases independent of coexisting risk factors have been reported in studies of fingolimod (0.4% with 0.5 mg/d), and siponimod (2% with 2 mg/d) (Kappos et al., 2018). Macular edema occurred at a low rate (0.3%–0.4%) of ozanimod in a pooled analysis of data from SUNBEAM and Phase 3 trial data, and all confirmed cases had predisposing comorbid conditions consisting of a history of macular oedema, macular oedema secondary to ocular trauma, and a history of retinopathy and optic neuritis (Selmaj et al., 2021). Therefore, ophthalmological examination is recommended before initiating treatment with an S1PR modulator owing to the risk of macular oedema.

There is a growing concern that patients treated with S1PR modulators may experience an increased risk of lymphopenia. Since all S1PR modulators link to S1PR1, the gradient of lymphocytes between peripheral lymph organs and blood altered by all of them. By this pathway, their main biological effects are obtained, with all their desired therapeutic implications (Kappos et al., 2021; Dumitrescu et al., 2023). Fingolimod does not interfere with lymphocyte activation. The effect of fingolimod on circulating lymphocytes is dose-dependent, decreasing by 20%–30% within the first week of treatment, with a recovery of the normal range about 1–2 months after treatment stop. Like fingolimod, siponimod induces lymphopenia by sequestration in lymph nodes. Siponimod reduces the peripheral lymphocyte counts to 20%–30% of baseline values within 4–6 h, with a recovery of the normal range with 10 days after treatment discontinuation in 90% of the patients. Studies confirmed that lymphocyte restoration after discontinuation are differences among S1PR modulators, being 6 weeks, 1–10 days, and 2–3 days for fingolimod, siponimod and ozanimod, respectively. Of note, the new S1PR modulators have a shorter half-life than that of fingolimod, which facilitates to faster lymphocyte restoration after discontinuation (Sanna et al., 2004; Baldin and Lugaresi, 2020; Kruger, Valenzuela, Thompson, Ouwerkerk-Mahadevan & Ruixo, 2023). In an EXPAND study, the grade 4 lymphopenia (<200/mmc) occurred in only 1% of patients treated with siponimod (Kappos et al., 2018). Furthermore, a SPMS patient developed severe lymphopenia 1 month after switching from fingolimod to siponimod, but the cumulative effects of fingolimod to siponimod on lymphocyte count cannot rule out (Sparaco et al., 2021).

It is worth noting that some AEs of S1PR modulators were fatal, which was also an important concern in clinical practice. In line with previous studies, infections were the most significant SOCs for both fingolimod and siponimod according to the FAERS database death reports. Serious infections including cryptococcal meningoencephalitis, neuro cryptococcosis, and progressive multifocal leukoencephalopathy (PML) were among the top 20 AEs associated with fingolimod treatment. On the other hand, sepsis, pneumonia, pyelonephritis, urosepsis, and PML ranked in the top 20 AE signals related to siponimod. PML is a chronic demyelinating disorder of the CNS caused by John Cunningham virus (JCV), which is ubiquitous virus that can become neurotropic and cause PML in rare patients with chronic cellular immunodeficiency (Cortese et al., 2021). Studies have reported that serum antibodies against JCV are present in approximately 50%–90% of the general population (Paz, Branco, Pereira, Spessotto & Fragoso, 2018). A reactivated infection potentially leading to PML occurs nearly exclusively in immunocompromised individuals, thus making MS patients taking immunosuppressive drug therapy a particularly at-risk group (Bohra et al., 2017; Sriwastava et al., 2021). Many cases of PML have been reported as a complication of DMT, including fingolimod (Nakahara et al., 2019), but the risk is 1:10,000 patients treated (Pérez-Jeldres et al., 2021). There were no serious opportunistic infections in ozanimod recipients. However, one case of PML in association with ozanimod treatment in a clinical trial was also identified (Sriwastava et al., 2022).

Patients with multiple sclerosis are vulnerable to the presence of potential drug-drug interactions (pDDIs) as they take numerous medications to treat MS, associated symptoms and comorbidities (Frahm et al., 2021). In the present study, among S1PR modulators-related AE reports, there were 30,321 (33%) cases in combination with other drugs. The top 20 concomitant drugs are shown in Supplementary Table S5. Overall, the most common types of concomitant drugs were other disease-modifying drugs (DMDs) (e.g., Tecfidera, ofatumumab, natalizumab, glatiramer acetate) and medication for symptom reduction (e.g., baclofen), followed by antidepressants (e.g., citalopram, venlafaxine, sertraline), sedative-hypnotics (e.g., clonazepam), antiepileptics (e.g., gabapentin, pregabalin), antipyretic analgesics (e.g., acetaminophen), hormonal system (e.g., levothyroxine sodium, prednisolone), antidiabetic (e.g., metformin), and antihypertensive (e.g., amlodipine besylate) medications. To maintain quality of life and improve functional outcomes, many patients seek additional help in the use of complementary and alternative medicines (CAM) such as vitamin D supplements. Studies have suggested that the combined use of DMDs, symptomatic therapeutics, comorbidity drugs and CAM lead to adverse drug effects that may have serious consequences for the patients. Debus et al. demonstrated that most common severe pDDIs occurred between citalopram and fingolimod (Debus et al., 2022). Citalopram is a selective serotonin reuptake inhibitor (SSRI), which is often prescribed to patients with anxiety disorders or depression. A side effect of citalopram may cause QT prolongation, which may lead to ventricular arrythmias or sudden cardiac death (Maljuric et al., 2015). Administration of fingolimod in the first dose may also prolong the QT interval, especially when given concomitantly with SSRIs. Thus, citalopram should be avoided within the first days after the start of fingolimod therapy, but afterwards there are no safety concerns so far (Bermel et al., 2015). Additionally, it was exhibited that methylprednisolone, acetylsalicylic acid and ibuprofen were the top triggers of pDDIs in patients with MS (Pöllmann and Feneberg, 2008), while most pDDIs were mild.

Although this study showed a potentially insightful relationship between the use of S1PR modulators and reporting of AEs in the real-world using FAERS database, it has some limitations. Firstly, the FAERS database is a spontaneous reporting system (SRS), and only observed AEs are registered, which might result in underreporting and reporting bias (Noguchi et al., 2021). Hartnell et al. demonstrated that the Weber effect occurred if the number of AE reports following the drug approval generally tends to diminish over time after a transient rise immediately after marketing (Hartnell and Wilson, 2004). Consistent with the previous study, both siponimod and ozanimod analyzed in this study demonstrated the Weber effect. Most S1PR modulators are new drugs with limited experience of post-market use, making our analysis prone to temporal bias. Secondly, disproportionality analysis of spontaneous reporting was used in the present study, including reporting odds ratio (ROR) and its 95% confidence interval to identify the statistical association between drugs and AEs. However, it may be influenced by notoriety bias, in which the number of reported AEs on a topic increases overall (Pariente, Gregoire, Fourrier-Reglat, Haramburu & Moore, 2007). The notoriety bias could also create a ripple effect, altering the reporting balance of other drugs associated with the same effect. Finally, due to the short marketing time, our study only analyzed and compared the AE signals of fingolimod, siponimod and ozanimod. A comprehensive investigation is necessary in the future study for the more recent S1PR modulator, ponesimod.

In conclusion, the present study identified various AE signals after the use of S1PR modulators based on real-world data from FAERS. We conducted a comprehensively study and discovered that the commonly reported potential AEs were increased risk of ocular events and nervous system disorders. In terms of fatality, progressive multifocal leukoencephalopathy was found to be strongly associated with both fingolimod and siponimod. Unexpected AEs, such as thrombosis as well as reproductive system and breast disorders, might also occur. However, there were variations in the potential IMEs frequently reported by S1PR modulators. Cardiac disorders and macular oedema were frequently reported with fingolimod, while malignancy was often reported related to fingolimod and siponimod. Additionally, infections were mostly reported to be associated with siponimod and ozanimod. Since the new generation of S1PR modulators have been used for a relatively short time after marketing compared to fingolimod, further research using various real-world database is necessary to find potential AEs related to S1PR modulators with MS.

## Data Availability

The original contributions presented in the study are included in the article/Supplementary Material, further inquiries can be directed to the corresponding authors.

## Abbreviations

S1P, Sphingosine-1-phosphate; MS, Multiple sclerosis; AE, Adverse event; IME, Important medical event; FDA, the US Food and Drug Administration; FAERS, the FDA Adverse Event Reporting System; ROR, Reporting odds ratio; PRR, Proportional reporting ratio, BCPNN, Bayesian confidence propagation neural network of information components; PT, Preferred term; SOC, system organ classes; RRMS, relapsing-remitting multiple sclerosis; SPMS, secondary progressive multiple sclerosis; PML, progressive multifocal leukoencephalopathy.
