# Supplementary material for: Potential adverse events associated with sphingosine-1-phosphate (S1P) receptor modulators in patients with multiple sclerosis: an analysis of the FDA adverse event reporting system (FAERS) database
Source: Front Pharmacol. 2024 May 23;15:1376494. doi: 10.3389/fphar.2024.1376494 (PMC11153721; doi:10.3389/fphar.2024.1376494)
Supplement: Supplementary file 1 [file DataSheet1.pdf]

Potential adverse events associated with sphingosine-1-phosphate (S1P) receptor modulators in patients with multiple sclerosis: an analysis of the FDA Adverse Event Reporting System (FAERS) database

Supplementary Table S1. Calculation formulas and judgment criteria of signal analysis methods

Method	Calculation formulars	Judgement criteria
ROR	ROR=$(a/c)/(b/d)$<br>95%CI=$e^{\ln(ROR)\pm1.96\sqrt{(\frac{1}{a}+\frac{1}{b}+\frac{1}{c}+\frac{1}{d})}}$	a⩾3, 95%CI>1
PRR	PRR=$a(c+d)/c(a+b)$<br>$\chi^2=[(ad-bc)^\wedge2](a+b+c+d)/[(a+b)(c+d)(a+c)(b+d)]$	a⩾3, PRR⩾2, χ^2⩾4
BCPNN	IC=$log2a(a+b+c+d)(a+c)(a+b)$<br>95%CI=$e^{\ln(IC)\pm1.96\sqrt{(\frac{1}{a}+\frac{1}{b}+\frac{1}{c}+\frac{1}{d})}}$	$IC_{025} > 0$

Abbreviations: a, the number of reports with suspect adverse drug event (ADE) of the suspect drug; b: the number of reports with the suspect ADE of all other drugs; c: the number of reports with all other ADEs of the suspect drug; d: the number of reports with all other ADEs of all other drugs; ROR, Reporting Odds Ratio; PRR, Proportional Reporting Ratio; CI: Confidence Interval; χ2: Chi-squared; BCPNN: Bayesian Confidence Propagation Neural Network; IC: Information Component; IC_{025}: the lower limit of the 95% two-sided CI of the IC.

Supplementary Table S2. Distribution of System Organ Classes (SOCs) for potential AEs of S1PR modulators.

SOCs	Fingolimod	Siponimod	Ozanimod	Total
Nervous system disorders	59383 (21.48)	4529 (21.60)	1981 (18.44)	65893 (21.38)
General disorders and administration site conditions	41794 (15.12)	4001 (19.08)	2035 (18.94)	47830 (15.52)
Investigations	32156 (11.63)	1708 (8.14)	796 (7.41)	34660 (11.25)

Musculoskeletal and connective tissue disorders	17076 (6.18)	1272 (6.07)	755 (7.03)	19103 (6.20)
Injury, poisoning and procedural complications	15467 (5.60)	1775 (8.46)	579 (5.39)	17821 (5.78)
Infections and infestations	15296 (5.53)	1300 (6.20)	631 (5.87)	17227 (5.59)
Gastrointestinal disorders	14995 (5.42)	1223 (5.83)	132 (12.30)	16350 (5.31)
Psychiatric disorders	14302 (5.17)	809 (3.86)	474 (4.41)	15585 (5.06)
Eye disorders	13778 (4.98)	850 (4.05)	277 (2.58)	14905 (4.84)
Respiratory, thoracic and mediastinal disorders	9371 (3.39)	590 (2.81)	365 (3.40)	10326 (3.35)
Skin and subcutaneous tissue disorders	7770 (2.81)	546 (2.60)	324 (3.02)	8640 (2.80)
Cardiac disorders	6575 (2.38)	371 (1.77)	161 (1.50)	7107 (2.31)
Neoplasms benign, malignant and unspecified	5265 (1.90)	333 (1.59)	100 (0.93)	5698 (1.85)
Vascular disorders	3947 (1.43)	439 (2.09)	277 (2.58)	4663 (1.51)
Renal and urinary disorders	3613 (1.31)	245 (1.17)	141 (1.31)	3999 (1.30)
Blood and lymphatic system disorders	3602 (1.30)	258 (1.23)	111 (1.03)	3971 (1.29)
Metabolism and nutrition disorders	2628 (0.95)	142 (0.68)	115 (1.07)	2885 (0.94)
Immune system disorders	1922 (0.70)	145 (0.69)	66 (0.61)	2133 (0.69)
Ear and labyrinth disorders	1774 (0.64)	117 (0.56)	52 (0.48)	1943 (0.63)
Reproductive system and	1536 (0.56)	55 (0.26)	56 (0.52)	1647 (0.53)

breast disorders				
Hepatobiliary disorders	1489 (0.54)	88 (0.42)	28 (0.26)	1605 (0.52)
Pregnancy, puerperium and perinatal conditions	1371 (0.50)	9 (0.04)	33 (0.31)	1413 (0.46)
Congenital, familial and genetic disorders	380 (0.14)	9 (0.04)	1 (0.01)	390 (0.13)
Endocrine disorders	320 (0.12)	13 (0.06)	11 (0.10)	344 (0.11)
Product issues	282 (0.10)	87 (0.41)	13 (0.12)	382 (0.12)
Surgical and medical procedures	217 (0.08)	45 (0.21)	24 (0.22)	286 (0.09)
Social circumstances	127 (0.05)	13 (0.06)	15 (0.14)	155 (0.05)
Total	276436 (100)	20972 (100)	10742 (100)	308150 (100)

Supplementary Table S3. Comparison of the AE reports of S1PR modulators at the system organ class (SOC) level by racial/ethnic group.

SOC	Fingolimod			Siponimod			Ozanimod		
	White	Black	Asian	White	Black	Asian	White	Black	Asian
Blood and lymphatic system disorders	2706	200	167	258	6	13	147	0	0
Cardiac disorders	4271	238	169	308	7	20	209	1	0
Congenital, familial and genetic disorders	287	21	17	9	0	0	2	0	0
Ear and labyrinth disorders	984	85	31	85	1	4	57	0	0
Endocrine disorders	186	24	5	13	0	2	13	0	0
Eye disorders	7920	776	245	700	17	20	356	0	1
Gastrointestinal disorders	8622	597	255	930	17	18	1583	0	6
General disorders and	22072	2014	671	2938	109	130	2531	0	0

administration site conditions									
Hepatobiliary disorders	928	78	124	62	1	24	37	0	0
Immune system disorders	1041	186	30	102	4	2	87	0	0
Infections and infestations	9453	628	364	1144	40	64	747	0	8
Injury, poisoning and procedural complications	7852	741	368	1345	65	47	733	0	1
Investigations	19901	1463	1137	1330	41	144	927	0	1
Metabolism and nutrition disorders	1550	197	58	113	5	7	135	0	0
Musculoskeletal and connective tissue disorders	9083	1045	287	980	49	27	884	0	0
Neoplasms benign, malignant and unspecified (incl cysts and polyps)	4151	124	178	338	3	28	122	0	4
Nervous system disorders	30744	3344	1526	3466	117	162	2411	0	2
Pregnancy, puerperium and perinatal conditions	948	55	71	12	4	1	43	0	0
Product issues	205	36	5	68	7	15	19	0	0
Psychiatric disorders	7193	711	178	627	29	14	583	0	0
Renal and urinary disorders	2264	222	103	215	8	2	169	0	0
Reproductive system	967	91	44	36	4	0	65	0	0

and breast disorders									
Respiratory, thoracic and mediastinal disorders	5376	506	172	466	25	18	442	0	0
Skin and subcutaneous tissue disorders	4641	345	141	414	9	11	406	0	2
Social circumstances	87	8	4	11	0	0	23	0	0
Surgical and medical procedures	224	0	1	51	0	0	49	0	0
Vascular disorders	2244	281	80	348	7	8	328	0	1

Supplementary Table S4. Top 20 potentially important medical adverse events (IMEs) of S1PR modulators used for multiple sclerosis.

**SOC**	**IME**	**N**	**PRR (χ^2)**	**ROR (95% CI)**	**IC**
Fingolimod					
Cardiac disorders	Bradycardia	1089	4.85 (3224.70)*	4.86 (4.58-5.16)*	2.24*
Nervous system disorders	Seizure	964	1.34 (82.41)	1.34 (1.26-1.43)*	0.42*
Eye disorders	Macular oedema	865	47.27 (29986.50)*	47.42 (43.93-51.17)*	5.19*
Nervous system disorders	Optic neuritis	684	18.44 (10080.83)*	18.49 (17.08-20.02)*	4.05*
Infections and infestations	Pneumonia	653	0.42 (532.57)	0.42 (0.39-0.45)	-1.26
Blood and lymphatic system disorders	Leukopenia	604	2.90 (737.14)*	2.90 (2.68-3.14)*	1.52*

Nervous system disorders	Loss of consciousness	585	1.10 (5.20)	1.10 (1.01-1.19)*	0.14*
Nervous system disorders	Hemiparesis	567	7.70 (3149.19)*	7.71 (7.09-8.39)*	2.88*
General disorders and administration site conditions	Death	488	0.12 (3233.95)	0.12 (0.11-0.13)	-3.06
Nervous system disorders	Syncope	458	1.04 (0.68)	1.04 (0.95-1.14)	0.06
Neoplasms benign, malignant and unspecified	Breast cancer	446	0.96 (0.86)	0.96 (0.87-1.05)	-0.06
Eye disorders	Blindness	399	2.28 (282.98)*	2.28 (2.07-2.52)*	1.18*
Neoplasms benign, malignant and unspecified	Basal cell carcinoma	382	5.49 (1356.11)*	5.50 (4.97-6.09)*	2.42*
Cardiac disorders	Myocardial infarction	358	0.50 (184.16)	0.49 (0.45-0.55)	-1.01
Nervous system disorders	Cerebrovascular accident	342	0.48 (197.98)	0.47 (0.43-0.53)	-1.07
Blood and lymphatic system disorders	Neutropenia	254	0.44 (184.25)	0.44 (0.39-0.49)	-1.19
Immune system disorders	Immunodeficiency	253	4.11 (578.96)*	4.11 (3.63-4.65)*	2.01*
Nervous system disorders	Epilepsy	238	1.85 (91.16) *	1.85 (1.62-2.10) *	0.88*
Neoplasms benign, malignant and unspecified	Malignant melanoma	234	3.74 (457.86) *	3.74 (3.28-4.26) *	1.88*
Cardiac disorders	Arrhythmia	234	1.14 (4.00) *	1.14 (1.00-1.30) *	0.19

**Siponimod**					
Nervous system disorders	Seizure	109	1.99 (54.01)	2.00 (1.66-2.41) *	0.99*
General disorders and administration site conditions	Death	101	0.33 (142.89)	0.32 (0.27-0.39)	-1.62
Neoplasms benign, malignant and unspecified	Basal cell carcinoma	73	13.53 (841.78) *	13.57 (10.78-17.09) *	3.75*
Infections and infestations	Pneumonia	60	0.51 (28.86)	0.51 (0.39-0.65)	-0.98
Eye disorders	Macular oedema	51	28.68 (1343.79) *	28.75 (21.80-37.91) *	4.82*
Nervous system disorders	Cerebrovascular accident	50	0.92 (0.36)	0.92 (0.70-1.21)	-0.12
Blood and lymphatic system disorders	Leukopenia	46	2.88 (56.25) *	2.88 (2.16-3.85) *	1.52*
Cardiac disorders	Bradycardia	45	2.58 (43.42) *	2.58 (1.93-3.46) *	1.36*
Infections and infestations	Sepsis	39	1.04 (0.06)	1.04 (0.76-1.42)	0.05
Nervous system disorders	Syncope	37	1.11 (0.38)	1.11 (0.80-1.53)	0.15
Nervous system disorders	Loss of consciousness	36	0.89 (0.48)	0.89 (0.64-1.24)	-0.17
Nervous system disorders	Hemiparesis	33	5.68 (126.79) *	5.68 (4.04-8.00) *	2.50*
Psychiatric disorders	Hallucination	32	1.39 (3.44)	1.39	0.47

				(0.98-1.96)	
Nervous system disorders	Transient ischaemic attack	31	2.91 (38.73) *	2.91 (2.04-4.14) *	1.54*
Cardiac disorders	Myocardial infarction	30	0.55 (11.17)	0.55 (0.38-0.78)	-0.87
Neoplasms benign, malignant and unspecified	Breast cancer	27	0.76 (1.98)	0.76 (0.52-1.11)	-0.39
Nervous system disorders	Optic neuritis	25	8.02 (152.93) *	8.02 (5.42-11.89) *	3.00*
Eye disorders	Cataract	25	1.26 (1.31)	1.26 (0.85-1.86)	0.33
Cardiac disorders	Atrial fibrillation	24	0.73 (2.39)	0.73 (0.49-1.09)	-0.45
Eye disorders	Blindness	21	1.57 (4.34) *	1.57 (1.02-2.41)	0.65
**Ozanimod**					
Nervous system disorders	Seizure	31	1.11 (0.32)	1.11 (0.78-1.57)	0.15
Infections and infestations	Pneumonia	31	0.51 (14.52)	0.51 (0.36-0.73)	-0.97
General disorders and administration site conditions	Death	29	0.18 (107.59)	0.18 (0.13-0.26)	-2.45
Nervous system disorders	Cerebrovascular accident	20	0.72 (2.32)	0.72 (0.46-1.11)	-0.48
Blood and lymphatic system disorders	Leukopenia	19	2.32 (14.24) *	2.32 (1.48-3.64) *	1.21 *
Eye disorders	Blindness	19	2.77 (21.54) *	2.78 (1.77-4.36) *	1.47 *

Renal and urinary disorders	Nephrolithiasis	19	2.40 (15.53) *	2.40 (1.53-3.77) *	1.26 *
Nervous system disorders	Neuropathy peripheral	18	1.10 (0.15)	1.10 (0.69-1.74)	0.13
Nervous system disorders	Optic neuritis	17	10.63 (147.94) *	10.65 (6.61-17.14) *	3.41 *
Nervous system disorders	Loss of consciousness	16	0.77 (1.07)	0.77 (0.47-1.26)	-0.37
Nervous system disorders	Transient ischaemic attack	15	2.74 (16.63) *	2.75 (1.66-4.56) *	1.46 *
Cardiac disorders6	Cardiac flutter	13	11.70 (126.80) *	11.71 (6.79-20.19) *	3.54 *
Respiratory, thoracic and mediastinal disorders	Pulmonary embolism	13	0.78 (0.80)	0.78 (0.45-1.34)	0.54
Cardiac disorders	Bradycardia	13	1.45 (1.83)	1.45 (0.84-2.50)	-0.36
Vascular disorders	Thrombosis	12	0.86 (0.29)	0.86 (0.49-1.51)	-0.22
Eye disorders	Macular oedema	11	11.95 (110.04) *	11.96 (6.62-21.62) *	3.57 *
Cardiac disorders	Arrhythmia	10	1.25 (0.51)	1.25 (0.67-2.33)	0.32
Metabolism and nutrition disorders	Diabetes mellitus	10	0.81 (0.45)	0.81 (0.44-1.51)	-0.30
Neoplasms benign, malignant and unspecified	Breast cancer	10	0.55 (3.64)	0.55 (0.30-1.03)	-0.86
Nervous system disorders	Hemiparesis	10	0.26 (11.61)	3.35 (1.80-6.24)	1.74 *

1.*Statistically significant association, i.e., the adverse events are detected as signals.

2. Abbreviations: SOC: System Organ Classes; IME: Important Medical Event; N: the number of reports of S1P receptor modulators-associates AEs; PRR: Proportional Reporting Ratio;χ^2: Chi-squared; ROR: Reporting Odds Ratio; CI: Confidence Interval; IC: Bayesian Confidence Propagation Neural Network of Information Components.

Supplementary Table S5 Top 20 concomitant drugs for S1PR modulators-related AEs from FAERS database.

**Fingolimod**		**Siponimod**		**Ozanimod**	
**Concomitant drugs**	**N**	**Concomitant drugs**	**N**	**Concomitant drugs**	**N**
Vitamin D	1000	Baclofen	67	Pprednisone	100
Dalfampridine	718	Dalfampridine	55	Gabapentin	93
Baclofen	563	Gabapentin	35	Vitamin D	71
Gabapentin	543	Vitamin D	33	Baclofen	57
Vitamin	375	Vitamin D3	31	Mesalamine	51
Ibuprofen	325	Acetaminophen	23	Vitamin D3	42
Pregabalin	260	Ofatumumab	21	Acetaminophen	30
Levothyroxine Sodium	230	Ibuprofen	20	Levothyroxine Sodium	28
Glatiramer Acetate	221	Pregabalin	20	Vitamins	27
Escitalopram	210	Amlodipine Besylate	19	Omeprazole	20
Sertraline	206	Vitamin B12	18	Alprazolam	19
Tecfidera	200	Vitamins	17	Lexapro	18
Vitamin B12	187	Aspirin	15	Aspirin	16
Acetaminophen	182	Duloxetine Hydrochloride	13	Ibuprofen	16
Clonazepam	159	Escitalopram	12	Budesonide	12
Citalopram Hydrobromide	158	Metformin	12	Sertraline	12
Levothyroxine	156	Venlafaxine	12	Pregabalin	12
Natalizumab	156	Sertraline	12	Lisinopril	12

Aspirin	155	Vitamin C	11	Tecfidera	11
Carbamazepine	153	Levothyroxine Sodium	11	Vitamin B12	10

Supplementary Table S6 Distribution of system organ classes (SOCs) for potential adverse events of ponesimod from the FAERS database.

SOC	N	ROR (95%CI)
Blood and lymphatic system disorders	10	1.27 (0.68-2.38)
Cardiac disorders	20	1.74 (1.11-2.73)
Congenital, familial and genetic disorders	1	0.70 (0.10-5.01)
Ear and labyrinth disorders	2	0.95 (0.24-3.80)
Endocrine disorders	1	0.81 (0.11-5.80)
Eye disorders	14	1.49 (0.87-2.53)
Gastrointestinal disorders	30	0.72 (0.50-1.04)
General disorders and administration site conditions	67	0.74 (0.57-0.96)
Hepatobiliary disorders	1	0.24 (0.03-1.69)
Immune system disorders	4	0.73 (0.27-1.96)
Infections and infestations	25	0.97 (0.65-1.46)
Injury, poisoning and procedural complications	49	0.94 (0.70-1.26)
Investigations	34	1.22 (0.86-1.73)
Musculoskeletal and connective tissue disorders	27	1.06 (0.72-1.57)
Neoplasms benign, malignant and unspecified (incl cysts and polyps)	12	0.88 (0.50-1.56)
Nervous system disorders	84	2.32 (1.84-2.94)
Pregnancy, puerperium and perinatal conditions	2	1.01 (0.25-4.06)
Product issues	4	0.49 (0.18-1.32)
Psychiatric disorders	17	0.62 (0.38-1.01)
Renal and urinary disorders	4	0.43 (0.16-1.14)
Reproductive system and breast disorders	3	0.70 (0.22-2.17)

Respiratory, thoracic and mediastinal disorders	36	1.64 (1.17-2.31)
Skin and subcutaneous tissue disorders	15	0.55 (0.33-0.92)
Surgical and medical procedures	13	2.03 (1.17-3.53)
Vascular disorders	9	0.90 (0.46-1.73)
